# Cutaneous and erosive oral lichen planus treated with upadacitinib

**DOI:** 10.1016/j.jdcr.2026.03.012

**Published:** 2026-03-13

**Authors:** Obarikanemi Nwogu, Kathleen Krivda, Aubrey Montebello, Jasmine Green, Ariel Gelman

**Affiliations:** a Transitional Year Internship, Walter Reed National Military Medical Center, Bethesda, Maryland; b Department of Dermatology, Walter Reed National Military Medical Center, Bethesda, Maryland

**Keywords:** cutaneous lichen planus, erosive lichen planus, genital lichen planus, JAK inhibitor, oral lichen planus, oral ulcers, skin of color, upadacitinib

## Introduction

Lichen planus (LP) is a chronic inflammatory disease affecting the skin, mucous membranes, hair, and nails. The mainstay of therapy for LP is topical or oral corticosteroids, and evidence-based guidance on systemic therapy for refractory disease is limited. Janus kinase (JAK) inhibitors pose a promising treatment for LP, particularly in cases that are refractory to conventional therapy, although evidence is limited to case reports and small series. We report a case of treatment-refractory oral, genital, and cutaneous LP treated successfully with off-label upadacitinib (Rinvoq).

## Case report

A 33-year-old African American female (Fitzpatrick type V) presented with a longstanding history of oral ulcers, genital ulcers, and nonhealing skin lesions, managed by rheumatology with presumed diagnoses of Bechet’s disease. Her medical history was notable for resolved drug-induced lupus to adalimumab. At the time of presentation, she was on 100 mg subcutaneous injection of anakinra daily for 4 months with minimal improvement in oral ulcers but no improvement in cutaneous lesions. She had previously trialed and failed hydroxychloroquine, prednisone, infliximab, adalimumab, tocilizumab 162 mg injection weekly, azathioprine 50 mg oral daily, tofacitinib 11 mg oral daily, tacrolimus 2 mg oral daily, and clobetasol 0.05% ointment.

**Fig 1:**
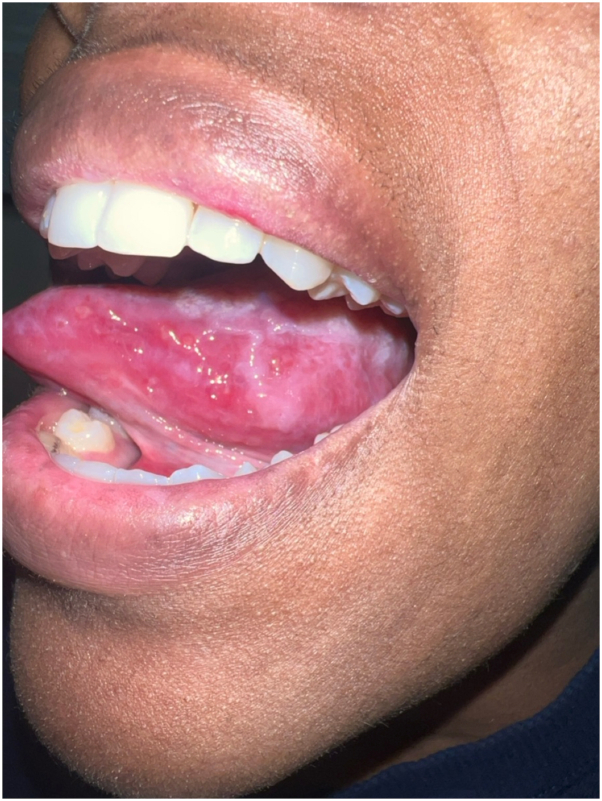
Numerous whitish plaques and ulcers on the left lateral tongue.

**Fig 2:**
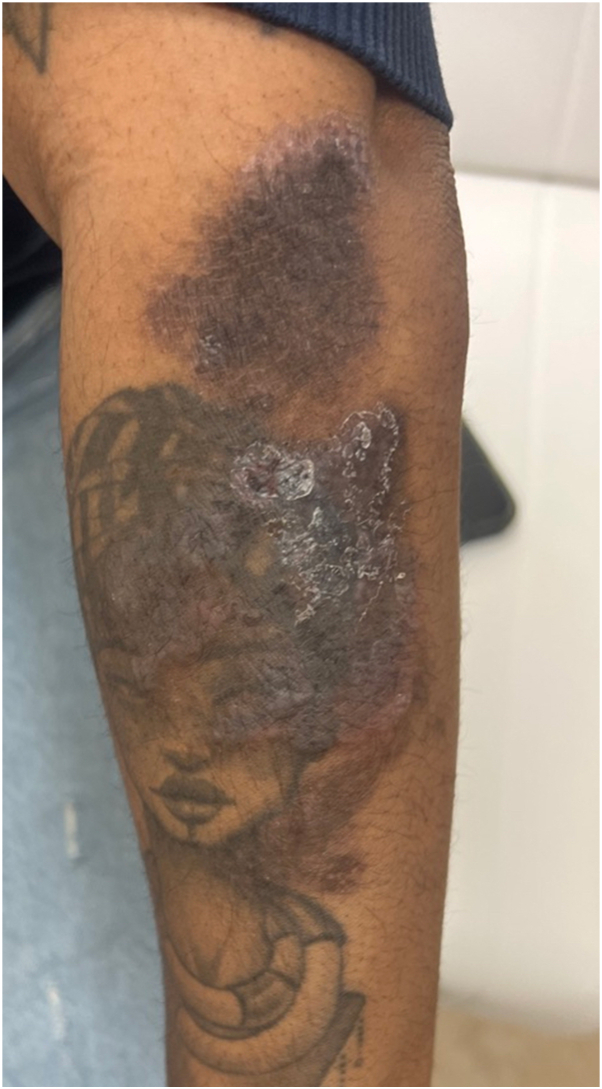
Flat-topped brown to violaceous plaques with overlying white scale on the left arm.

**Fig 3:**
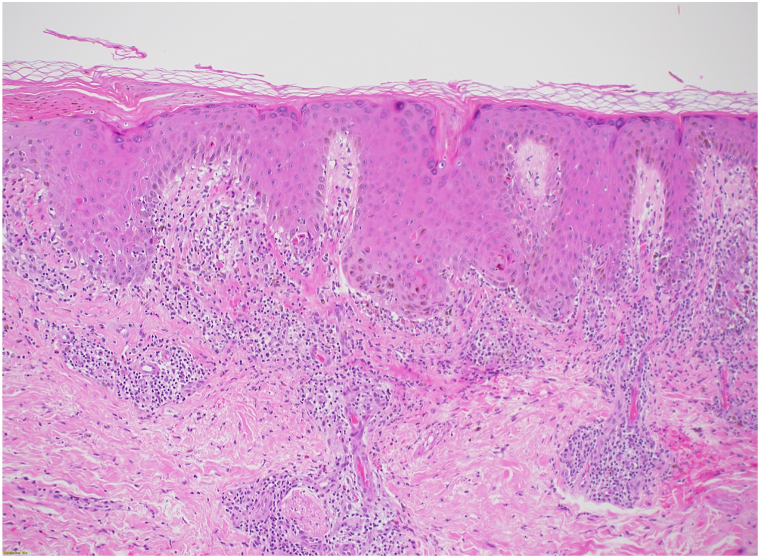
Lichenoid vacuolar interface dermatitis with dyskeratotic keratinocytes, pigment incontinence, and perivascular superficial and mid dermal lymphocytic infiltrate.

Her physical examination was notable for numerous violaceous to hyperpigmented plaques with overlying erosions on her upper extremities and multiple ulcers with Wickman striae on the lateral aspect of the tongue bilaterally (Figs 1 and 2). The vulvar examination was notable for erythema and well-demarcated erosions of the vaginal introitus. Mucosal biopsy was deferred due to patient preference. A 4-mm punch biopsy of a plaque on her arm revealed robust lichenoid vacuolar interface dermatitis with dyskeratotic keratinocytes, pigment incontinence, and perivascular superficial and mid dermal lymphocytic infiltrate (Fig 3). No vasculitis or significant mucin deposition was appreciated. Direct immunofluorescence was positive for IgM with cytoid bodies and linear deposition of fibrinogen along the dermoepidermal junction. Laboratory workup revealed negative antinuclear antibody, negative antihistone, negative anti-Sjogren's syndrome related anitbody A and B, and negative anti-dsDNA antibodies, with normal complete blood count and complete metabolic panel. Overall, her clinical findings and biopsy results were consistent with erosive oral and cutaneous LP, rather than Behcet’s disease. Lupus was also considered, given her history of drug-induced lupus and the sun-exposed distribution of the forearm rash; however, this diagnosis was less favored based on negative serologies, as well as histopathologic and direct immunofluorescence findings characteristic of LP.

Given the extensive nature of her disease and failure of multiple therapies, the decision was made to initiate systemic treatment with upadacitinb, a selective Janus kinase 1 (JAK1) inhibitor, at a dose of 30 mg daily. Topical corticosteroids and calcineurin inhibitors were continued as needed for breakthrough plaques. Baseline screening laboratory tests were unremarkable.

**Fig 4:**
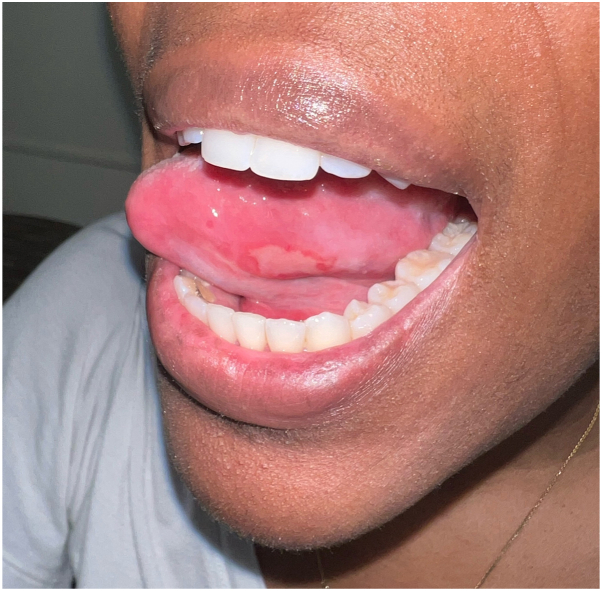
Significant improvement with residual shallow erosion on the left lateral tongue.

**Fig 5:**
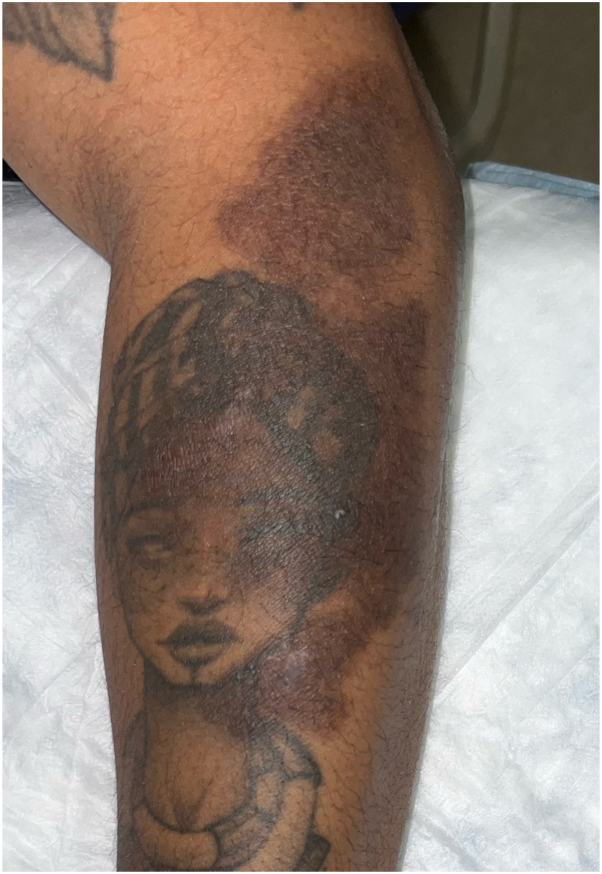
Postinflammatory hyperpigmentation of the left arm.

At follow-up 8 months after starting upadacitinib, the patient had resolution of cutaneous lesions and genital lesions and noted significant improvement of oral ulcers. She noted significant improvement of her skin within 3 weeks and improvement of her oral/genital ulcers within 6 to 8 weeks. She reported 1 to 3 small ulcers monthly, which were significantly less painful than prior and did not interfere with eating. The examination was notable for hyperpigmented patches consistent with postinflammatory hyperpigmentation on the upper extremities and shallow erosions on the lateral tongue (Figs 4 and 5). Regular laboratory monitoring remained within normal limits. Given her significant clinical improvement, treatment options—including tapering upadacitinib to 15 mg or continuing the 30 mg dose—were discussed with the patient. She elected to remain on 30 mg, with plans to reassess dose reduction at follow-up.

## Discussion

This case illustrates the successful treatment of erosive LP with Upadacitinib after failure of numerous therapies. JAK inhibitors block the JAK-STAT signaling pathway, which plays a crucial role in inflammatory processes. Upadacitinib selectively targets JAK1, which is involved in the signaling of multiple inflammatory cytokines implicated in LP pathogenesis.^1^^,^^2^ The pathogenesis of LP is incompletely understood but thought to involve the activation of cytotoxic T lymphocytes against basal keratinocytes. The inappropriately activated cytotoxic T cells are further influenced by Th1-type cytokines, including interferon-gamma (IFN-γ), interleukin-21 (IL-21), tumor necrosis factor-alpha (TNF-α), and IL-12.^3^ IFN-γ plays a central role by priming keratinocytes to increase their susceptibility to CD8+ T cell–mediated cytotoxicity through upregulation of major histocompatibility complex class I molecules, a process mediated via the JAK/STAT pathway. Other inflammatory pathways, including the interleukin 23/Th17 axis and IL-4, also play a role in the pathogenesis of LP.^1^ By inhibiting JAK1, upadacitinib disrupts signaling from IFN-γ, IL-21, IL-6, IL-4, and TNF- α.^3^

Current evidence supporting the use of upadacitinib in refractory LP is derived from observational studies. These reports have highlighted the potential of upadacitinib in treating refractory LP of various forms, including pediatric cutaneous LP, erosive oral LP, and erosive esophageal LP, with significant clinical improvement seen within 3 weeks in the majority of cases.^4^, ^5^, ^6^, ^7^, ^8^, ^9^, ^10^ In published case reports and small case series, upadacitinib has primarily been administered at 15 mg orally once daily across cutaneous, oral, and esophageal LP variants. Several patients demonstrated relatively rapid clinical improvement, specifically pruritus relief and decreased inflammation were observed within 2 weeks in cutaneous disease, with near resolution by 6 weeks, whereas erosive oral lesions and dysphagia improved within 4 to 12 weeks of therapy.^6^^,^^7^ A single case of esophageal LP was treated with 30 mg daily, with macroscopic and histological recovery within 3 months.^8^ In a case series of 7 patients with cutaneous LP, 5 treated with 15 mg daily achieved significant clearance and improved quality of life within days to weeks.^9^ Our patient’s timeline of improvement supports these findings.

This case contributes to the growing body of evidence supporting JAK inhibitors as a treatment for inflammatory dermatoses and reinforces the pathogenesis-based rationale for JAK1 inhibition in LP. Upadacitinib has also been used off-label with success in alopecia areata, hidradenitis suppurativa, vitiligo, and cutaneous and systemic lupus erythematous.^2^^,^^11^ Key considerations for clinical practice include appropriate patient selection for cases refractory to conventional therapies, careful risk-benefit assessment with thorough patient counseling, and ensuring close follow-up. Additionally, the burden of residual postinflammatory hyperpigmentation, particularly notable in this patient with a darker skin type, represents a common complication of LP that can addressed with both laser and medical therapy. Overall, case-based evidence suggests that upadacitinib can be employed for LP treatment. However, larger controlled studies are needed to establish optimal dosing, duration of therapy, and long-term safety profiles for LP patients.

## Conflicts of interest

None disclosed.
